# Prostate cancer metastases to the rectum: A case report

**DOI:** 10.1186/1477-7819-9-56

**Published:** 2011-05-21

**Authors:** Tariq O Abbas, Abdulla R Al-Naimi, Rafie A Yakoob, Issam A Al-Bozom, Abdulkader M Alobaidly

**Affiliations:** 1 Hamad General Hospital, Doha, Qatar

## Abstract

Prostate cancer rarely metastasis to the rectum. Findings in the patient reported here emphasize the importance of the relationship between urinary and gastrointestinal symptoms in detecting prostatic neoplasms in older male patients.

## Background

Prostate cancer has the potential to advance loco-regionally to adjacent organs. This spread can take place via different routes, including direct invasion and through lymphatic channels. It is very rare for prostate cancer to metastasize to nearby organs, including the rectum. We describe here a patient presenting with prostate cancer metastasizing to the rectum.

## Case report

A 60-year-old man was referred to our urology facility after experiencing severe weight loss (30 kg in 3 months) and bleeding from the rectum, together with upper abdominal pain and vomiting. Over the previous year, he had experienced painless hematuria and voiding difficulty.

**Figure 1:**
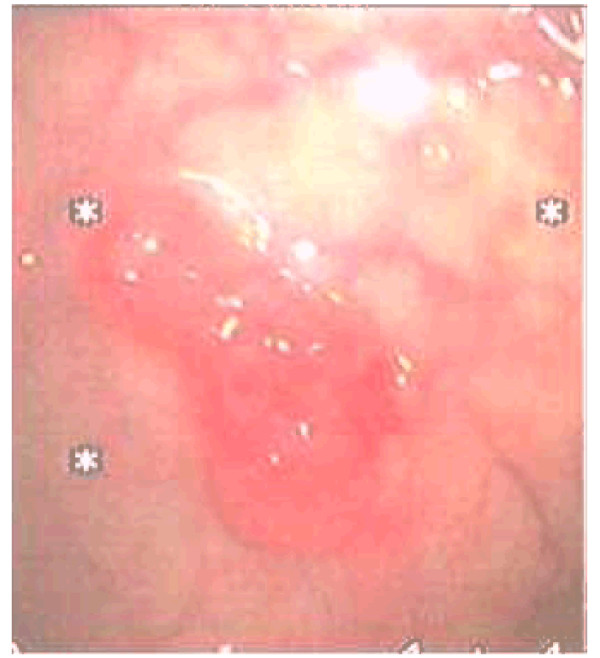
**Colonoscopy picture showing a distal rectal sessile mass lying about 15 cm from the anal verge**.

**Figure 2:**
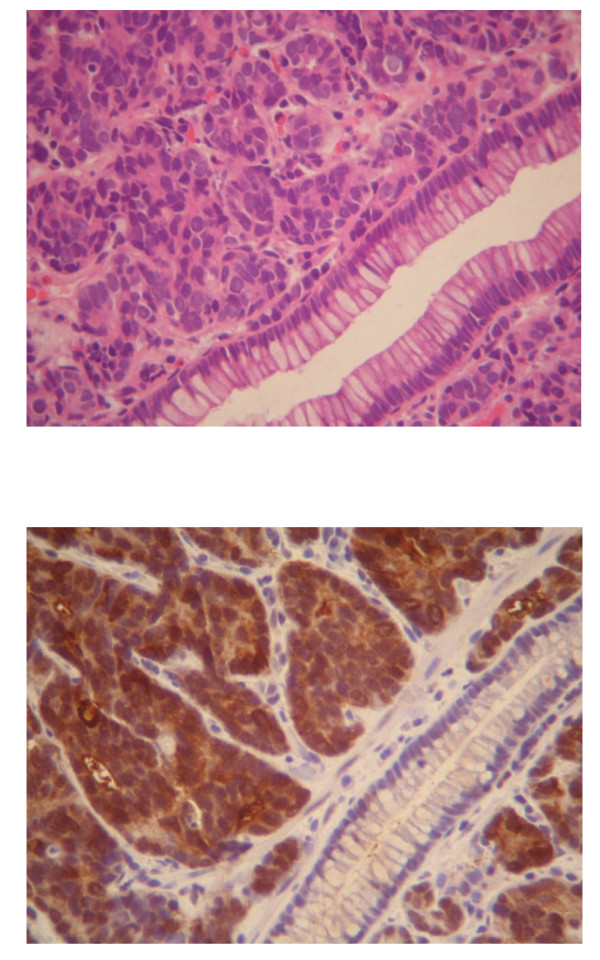
**(Top) Sections showing tumor acini adjacent to a normal colonic crypt (Hematoxylin & Eosin ×400); (Bottom) Staining of the same microscopic focus with anti-PSA antibody, showing that tumor cells were positive while normal colonic crypts were negative (immunohistochemistry-PSA ×400)**.

He was thoroughly examined, including undergoing a colonoscopy, which revealed a distal rectal sessile mass lying about 15 cm from the anal verge [Figure 1]. Histopathologic examination of the biopsy showed that it was a metastatic prostatic adenocarcinoma [Figure 2].

**Figure 3:**
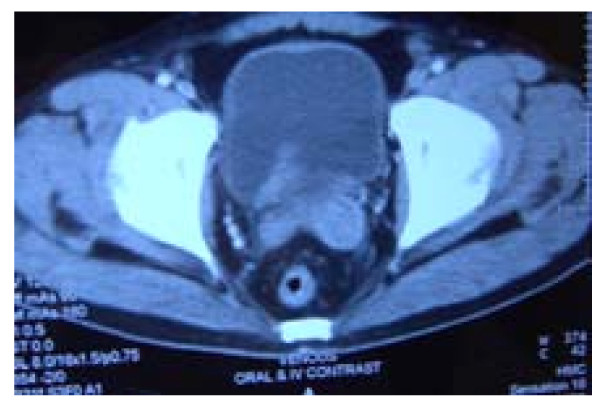
**CT scan of the abdomen showing the prostatic mass invading the urinary bladder wall and being separate from the rectum**.

**Figure 4:**
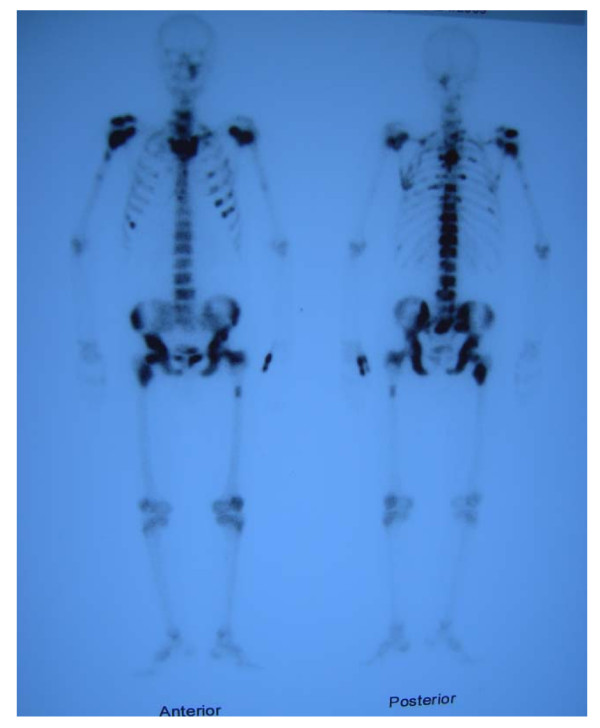
**TC-99 MDP bone scintigraphy showing widespread bone metastatic lesions**.

A CT scan of his abdomen showed that the prostatic mass had invaded the urinary bladder wall and that the biopsied mass was separate from the rectum [Figure 3]. His serum PSA concentration was high (983 ng/ml). In addition, TC-99 MDP bone scintigraphy showed widespread bone metastatic lesions [Figure 4].

He was started on hormone therapy and followed up by the oncology department.

## Discussion

Prostate cancer is a slowly growing neoplasm that can easily be missed during its early stages. Patients not previously diagnosed with prostatic adenocarcinoma may present initially with metastases [1]. In contrast, PSA may not be expressed in all patients with prostatic adenocarcinoma [2].

Prostate cancer extension to colorectal tissue can occur through at least 3 potential routes. The first is direct invasion through Denonvilliers fascia and infiltration into the rectum. The second is through lymphatics, since the prostate and rectum share some lymphatic drainage to groups of pelvic lymph node [3]. Third, prostate cancer cells can spread through needle biopsy, by seeding into peri-rectal or rectal tissue along the needle biopsy; this, route, however, is extremely rare [4,5].

Prostate cancer metastasis to the recto-sigmoid region can occur by subserosal metastatic implant of the malignant tissues [6]. The incidence of rectal infiltration by prostatic adenocarcinoma is extremely rare, being encountered on average once every two years by a busy colorectal practice [7].

## Conclusion

Findings in the patient reported here emphasize the importance of the relationship between urinary and gastrointestinal symptoms in detecting prostatic neoplasms in older male patients. Careful immunohistochemical examination of specimens can prevent major surgical interventions in favor of hormonal and radiological therapies.

## Consent

Written informed consent was obtained from the patient for publication of this case report and accompanying images. A copy of the written consent is available for review by the Editor-in-Chief of this journal.

## Competing interests

The authors declare that they have no competing interests.

## Authors' contributions

TA carried out the history, physical examination and the provisional draft.

AAN participitate in the sequence alignment and drafted the manuscript again. IAB carried out the histopathology.

RY carried out the colonoscopy and the re ctal biopsy.

AA involoved in the patient management and data collection.

All authors read and approved the final manuscript.
